# Supplementary figures and images for: Direct and legacy effects of plant-traits control litter decomposition in a deciduous oak forest in Mexico
Source: PeerJ. 2018 Jun 29;6:e5095. doi: 10.7717/peerj.5095 (PMC6027662; doi:10.7717/peerj.5095)

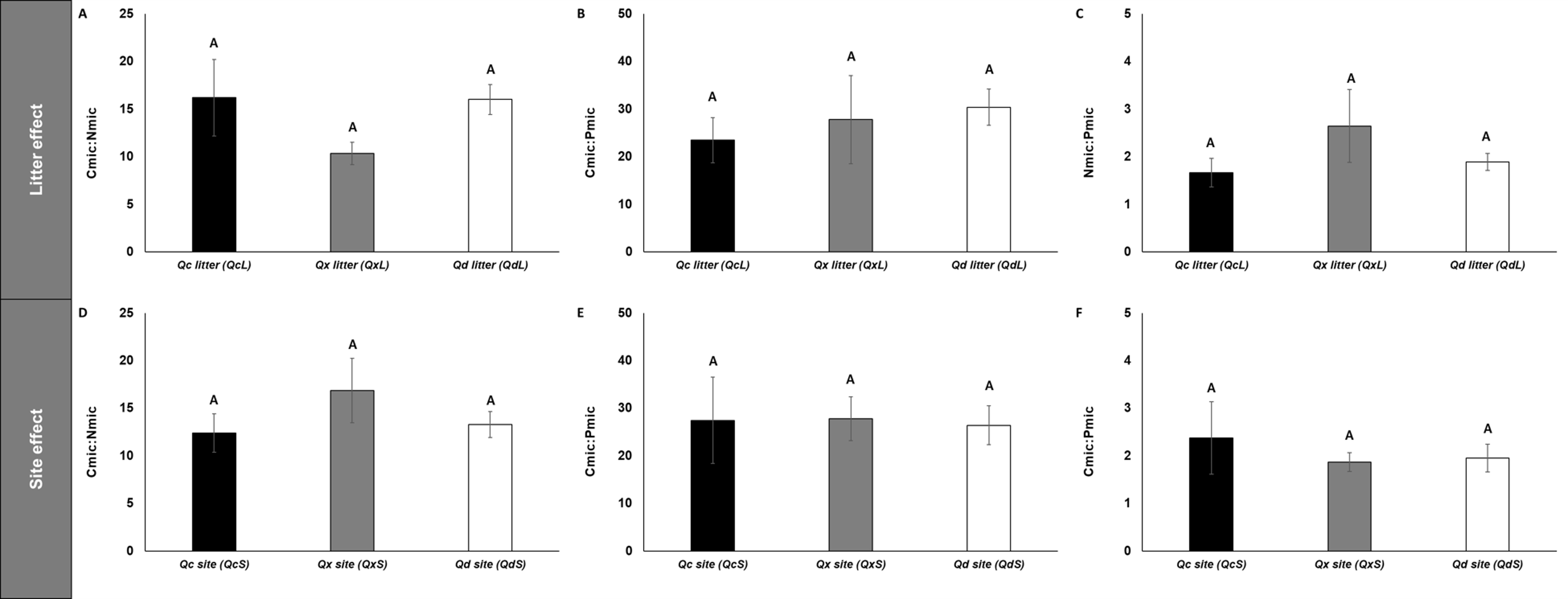

Supplement: Figure S1 — Different uppercase letters indicate significant differences ( P < 0.05) between treatments. [file peerj-06-5095-s001.png]
